# Supplementary material for: Effectiveness and Safety of Ayurvedic Medicines in Type 2 Diabetes Mellitus Management: A Systematic Review and Meta-Analysis
Source: Front Pharmacol. 2022 Jun 8;13:821810. doi: 10.3389/fphar.2022.821810 (PMC9213670; doi:10.3389/fphar.2022.821810)
Supplement: Supplementary file 1 [file DataSheet1.zip › Supplementary Material/Forest and Funnel Plots/Punica granatum/HDL-C.pdf]

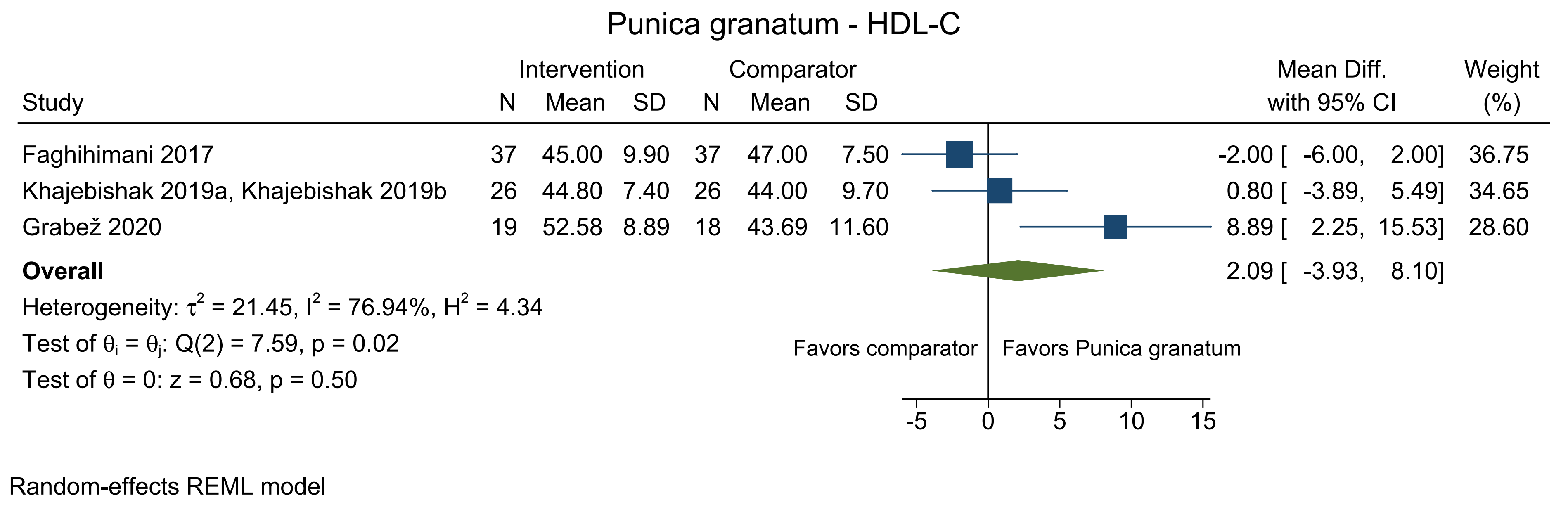
Punica granatum - HDL-C
Intervention
Comparator
Mean Diff.
Weight
Study
N
Mean
SD
N
Mean
SD
with 95% CI
(%)
Faghihimani 2017 37 45.00 9.90 37 47.00 7.50 -2.00 [-6.00, 2.00] 36.75
Khajebishak 2019a, Khajebishak 2019b 26 44.80 7.40 26 44.00 9.70 0.80 [-3.89, 5.49] 34.65
Grabež 2020 19 52.58 8.89 18 43.69 11.60 8.89 [2.25, 15.53] 28.60
Overall 2.09 [-3.93, 8.10]
Heterogeneity: τ² = 21.45, I² = 76.94%, H² = 4.34
Test of θi = θj: Q(2) = 7.59, p = 0.02
Test of θ = 0: z = 0.68, p = 0.50
Favors comparator
Favors Punica granatum
-5
0
5
10
15
Random-effects REML model
